# The Usefulness of Continuous Respiratory Sound Monitoring for the Detection of Pulmonary Atelectasis in a Ventilated Extremely Low Birth Weight Infant

**DOI:** 10.7759/cureus.65394

**Published:** 2024-07-25

**Authors:** Masashi Zuiki, Tatsuji Hasegawa, Shinichiro Ohshimo, Tomoko Iehara, Nobuaki Shime

**Affiliations:** 1 Department of Pediatrics, Graduate School of Medical Science, Kyoto Prefectural University of Medicine, Kyoto, JPN; 2 Department of Emergency and Critical Care Medicine, Hiroshima University Graduate School of Biomedical and Health Sciences, Hiroshima, JPN

**Keywords:** bronchoalveolar lavage, respiratory sounds, lung sound spectrograms, extremely low birth weight infants, pulmonary atelectasis

## Abstract

The assessment of auscultation using a stethoscope is unsuitable for continuous monitoring. Therefore, we developed a novel acoustic monitoring system that continuously, objectively, and visually evaluates respiratory sounds. In this report, we assess the usefulness of our revised system in a ventilated extremely low birth weight infant (ELBWI) for the diagnosis of pulmonary atelectasis and evaluation of treatment by lung lavage.

A female infant was born at 24 weeks of age with a birth weight of 636 g after emergency cesarean section. The patient received invasive mechanical ventilation immediately after birth in our neonatal (NICU). After obtaining informed consent, we monitored her respiratory status using the respiratory-sound monitoring system by attaching a sound collection sensor to the right anterior chest wall. On day 26, lung-sound spectrograms showed that the breath sounds were attenuated simultaneously as hypoxemia progressed. Finally, chest radiography confirmed the diagnosis as pulmonary atelectasis. To relieve atelectasis, surfactant lavage was performed, after which the lung-sound spectrograms returned to normal. Hypoxemia and chest radiographic findings improved significantly. On day 138, the patient was discharged from the NICU without complications. The continuous respiratory-sound monitoring system enabled the visual, quantitative, and noninvasive detection of acute regional lung abnormalities at the bedside. We, therefore, believe that this system can resolve several problems associated with neonatal respiratory management and save lives.

## Introduction

Pulmonary atelectasis, a complication in ventilated infants, is one of the most common causes of respiratory failure. Accurate and immediate diagnosis of pulmonary atelectasis is important for enabling appropriate treatment, including lung lavage, and improving prognosis. Although the diagnosis of pulmonary atelectasis is based on chest radiography (CXR), the disadvantages of CXR include the risks associated with radiation exposure and its infrequency [1].

Suggestive signs of atelectasis include localized reduced breath sounds as atelectasis is caused by complete airway obstruction [2]. Although clinicians use a stethoscope to aid in the diagnosis of respiratory disorders, auscultation assessment using a stethoscope is subject to inherent inter-observer variability and lacks objectivity. Additionally, it is impractical for neonatologists to continuously use a stethoscope during ventilator management in the neonatal ICU (NICU). To address this issue, we developed a novel monitoring system that continuously, objectively, and visually evaluates respiratory sounds. In brief, the system consists of a visual monitor that displays real-time respiratory sound spectrograms and sensors attached to the chest walls [3-6]. We present the first documented usefulness analysis of the respiratory sound monitoring system in a ventilated extremely low birth weight infant (ELBWI) for the diagnosis of pulmonary atelectasis and evaluation of treatment by lung lavage.

## Case presentation

**Figure 1:**
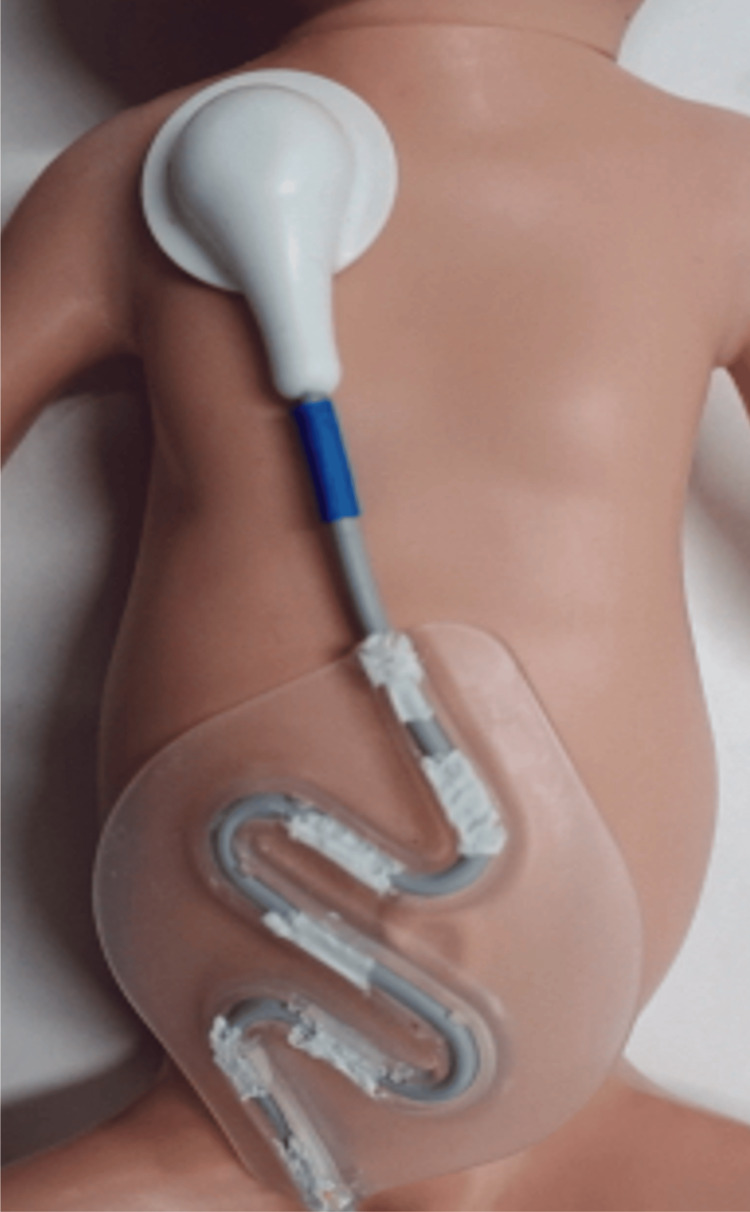
Sound collection sensors attached to the right chest walls of a manikin of the same size as our patient

The patient was a Japanese female with a birth weight of 636 g born at 24 weeks and 0 days of gestation via emergency cesarean delivery at another hospital. The mother was a healthy, primigravid, 28-year-old woman. The patient was the first twin in a monochorionic diamniotic twin pregnancy. The Apgar scores were 2 at one minute and 6 at five minutes. The infant was intubated immediately after birth, and surfactant was administered. Mechanical ventilation was required for respiratory distress syndrome. Although intravenous medications were administered to treat symptomatic patent ductus arteriosus (PDA) on days 1, 2, 21, and 22, the PDA could not be closed. On day 23, the newborn was transferred to our hospital for PDA surgery. Initially, ventilator support was provided using a synchronized intermittent mandatory ventilation mode at our hospital. After informed consent was obtained from the parents, we monitored her respiratory status using the respiratory-sound monitoring system by attaching a sound collection sensor to the right anterior chest (Figure 1).

**Figure 2:**
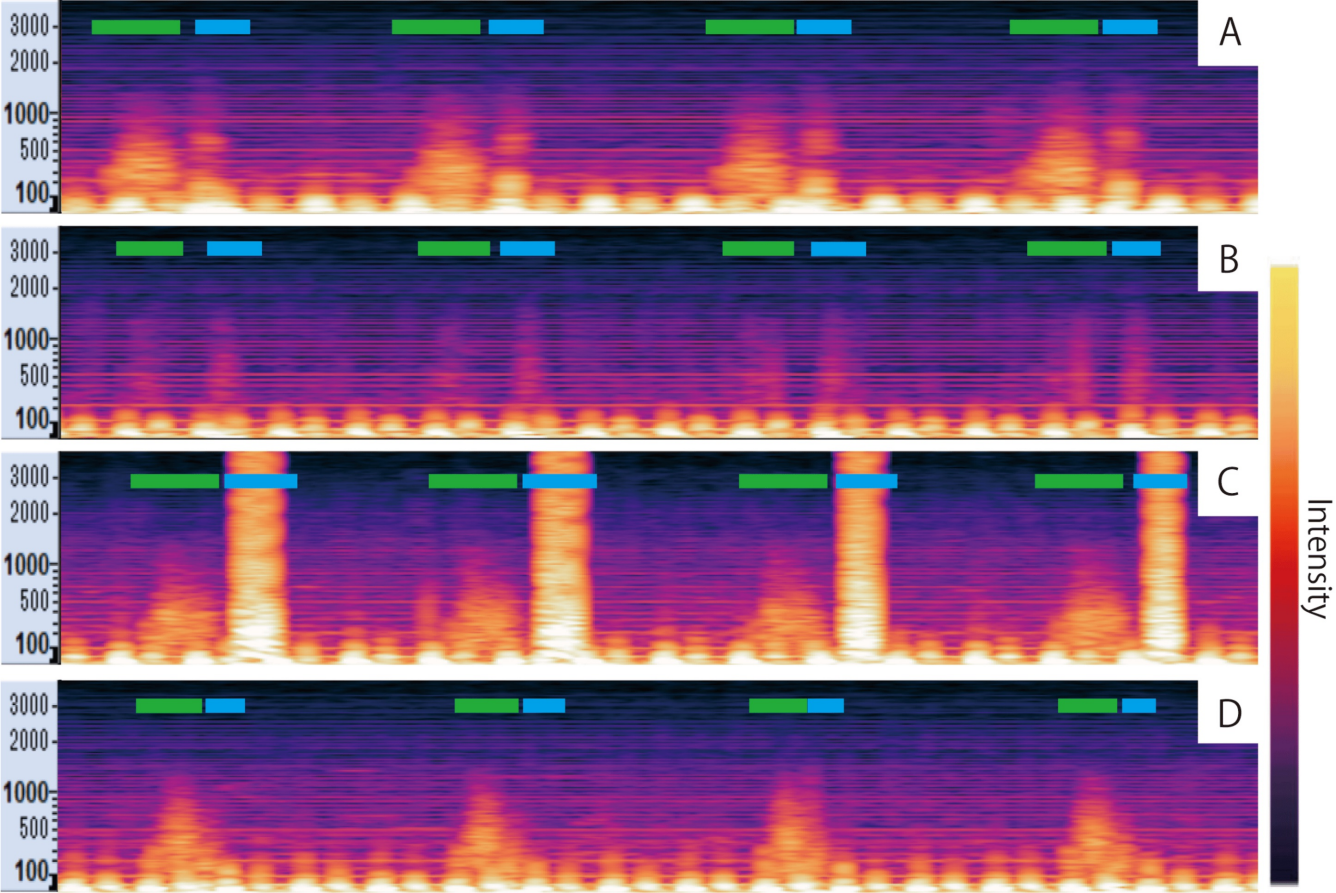
Sound spectrograms Sound spectrograms show time on the horizontal axis and frequency on the vertical axis, with amplitude (intensity) of respiratory sounds represented by brightness. Green bars indicate inspiration; blue bars indicate expiration. Admission sound spectrogram revealing normal lung sound pattern (A). Spectrograms suddenly showed a marked decrease in intensity when atelectasis developed (B). Immediately after lung lavage, high-intensity expiratory sounds were detected, which may reflect obstruction due to tracheal secretions (C). Three hours later, the abnormal sounds returned to normal (D)

**Figure 3:**
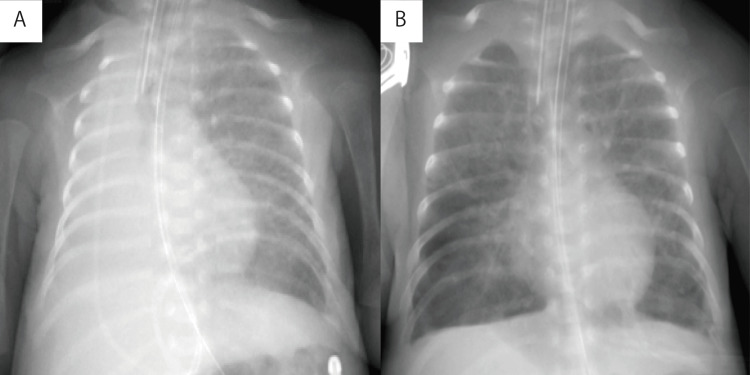
Chest radiographs Right pulmonary atelectasis (A) followed by the condition after surfactant lavage (B) was indicated

A sound spectrogram of normal lung sounds on admission is shown in Figure 2A. On day 26, the sudden decrease in intensity of a lung-sound spectrogram (Figure 2B) was followed by a decline in percutaneous oxygen saturation (SpO_2_), with an oxygenation index of 7.1. Pulmonary atelectasis was finally diagnosed using CXR (Figure 3A). To relieve the atelectasis, surfactant lavage was performed per a previous report [7]. A phospholipid surfactant (3 mL/kg) at a concentration of 5 mg/mL was instilled through the endotracheal tube, and aspiration was performed after gentle manual squeezing. This process was performed five times using positive-pressure ventilation. Immediately after the lung lavage, the breath sounds became audible as expiratory high-frequency sounds, which may reflect lower airway obstruction due to tracheal secretions (Figure 2C), and returned to normal three hours later (Figure 2D). Hypoxemia improved, with an oxygenation index of 2.0, and CXR findings showed that the atelectasis had disappeared (Figure 3B). Surgical PDA ligation was performed under general anesthesia on day 32. The patient’s operative course was uneventful. The patient was extubated on day 51 and discharged with complete recovery on day 138.

## Discussion

In this report, we demonstrated the utility of a respiratory-sound monitoring system for the diagnosis of pulmonary atelectasis and its treatment effect in a ventilated ELBWI. To the best of our knowledge, this is the first report to demonstrate the usefulness of continuous assessment of respiratory sounds in a ventilated immature infant. Recently, several studies on adults and children have shown that digital signal information of lung sounds serves as a reliable tool for diagnosing lung abnormalities and disorders [8-12]. However, electronic stethoscopes have limited use for continuous monitoring. Therefore, we developed a new respiratory sound monitoring system that can continuously visualize respiratory sounds at the bedside, contributing to the detection of pneumothorax and pleural effusion [3], prediction of complications after extubation [4], and upper airway assessment or quantification of aspiration risk during anesthesia care [5,6].

Our respiratory sound monitoring system has several clinical implications for use in the NICU. Primarily, despite advances in neonatal intensive care, respiratory monitoring methods such as pulse oximetry or capnometer use remain limited in clinical applications for neonates [13,14]. Electrical impedance tomography (EIT) is a new clinical imaging tool for monitoring the distribution of ventilation, but its utility in ELBWI is limited [15,16]. We believe that combining these methods with our sound monitoring system will improve the speed and accuracy of diagnosis because of the addition of local sound information. Next, neonatal respiratory conditions can be assessed objectively and continuously using our respiratory-sound monitoring system. Therefore, this system may detect atelectasis before it becomes severe. Notably, this system may have high sensitivity but low specificity for diagnosis. If a decrease in intensity in the lung spectrogram is detected, as in this case, chest radiographs should be performed to rule out not only atelectasis but also other possibilities such as a malpositioned endotracheal tube, pneumothorax, or pleural effusion.

The use of our system for newborns has a few limitations. Firstly, environmental noise in the NICU, such as the general background noise or that of the ventilator, may result in poor-quality chest sound recordings and inaccurate assessments. Despite recommendations for maintaining quietness in NICUs given the impact on the development of premature infants, these noise standards are often exceeded [17]. We suggest that staff training on noise reduction is required to monitor low-volume lung sounds in infants. In addition, internal body sounds, such as heart and bowel sounds, act as sources of noise in respiratory sound studies.

Although this system is specially designed in terms of materials and cable layout to prevent noise and is equipped with mechanical reduction technologies, such as sensor shape and damper structure, techniques to clearly distinguish lung sounds are needed for premature newborns, e.g., noise reduction based on signal processing. Next, this system can complicate nursing care for newborns with many devices because the sound collection sensor is slightly large for newborns with a small body and is not wireless. Innovations in this system are necessary for its clinical application in NICUs. Finally, the single-case study design of this report may have introduced biases. Studies with large sample sizes are needed to clarify the usefulness of continuous respiratory sound monitoring for immature infants.

## Conclusions

Based on our findings, a continuous respiratory sound monitoring system is useful for diagnosing pulmonary atelectasis and evaluating treatment by lung lavage in a ventilated ELBWI. The system can be used for visual, quantitative, and noninvasive monitoring in NICUs. We believe that this system can help resolve several problems associated with neonatal respiratory management.
